# A demonstration of the antimicrobial effectiveness of various copper surfaces

**DOI:** 10.1186/1754-1611-7-8

**Published:** 2013-03-27

**Authors:** Victor K Champagne, Dennis J Helfritch

**Affiliations:** 1 U.S. Army Research Laboratory, Aberdeen Proving Ground, MD, USA; 2 Dynamic Science, Aberdeen, MD, USA

## Abstract

**Background:**

Bacterial contamination on touch surfaces results in increased risk of infection. In the last few decades, work has been done on the antimicrobial properties of copper and its alloys against a range of micro-organisms threatening public health in food processing, healthcare and air conditioning applications; however, an optimum copper method of surface deposition and mass structure has not been identified.

**Results:**

A proof-of-concept study of the disinfection effectiveness of three copper surfaces was performed. The surfaces were produced by the deposition of copper using three methods of thermal spray, namely, plasma spray, wire arc spray and cold spray The surfaces were then inoculated with meticillin-resistant *Staphylococcus aureus* (MRSA). After a two hour exposure to the surfaces, the surviving MRSA were assayed and the results compared.

The differences in the copper depositions produced by the three thermal spray methods were examined in order to explain the mechanism that causes the observed differences in MRSA killing efficiencies. The cold spray deposition method was significantly more effective than the other methods. It was determined that work hardening caused by the high velocity particle impacts created by the cold spray technique results in a copper microstructure that enhances ionic diffusion, and copper ions are principally responsible for antimicrobial activity.

**Conclusions:**

This test showed significant microbiologic differences between coatings produced by different spray techniques and demonstrates the importance of the copper application technique. The cold spray technique shows superior anti-microbial effectiveness caused by the high impact velocity imparted to the sprayed particles which results in high dislocation density and high ionic diffusivity.

## Background

Bacterial growth on surfaces is a cause of concern in many hospitals and food processing industries due to the possibility of increased risk of bacterial infection [1]. The bacterial contamination of hospital surfaces, including patient rooms, nurse stations and kitchens has been extensively documented [2-5]. Contamination of meat and vegetable preparation surfaces, including refrigerators, and conveyors have also been the subject of investigation [6-10]. In addition to topically applied disinfectants, the use of surfaces that can self-disinfect would enhance overall infection prevention.

In the last few decades, work has been done on the antimicrobial properties of copper and its alloys against a range of micro-organisms threatening public health in food processing and healthcare applications [11]. The use of copper and copper alloys for frequently touched surfaces such as door and furniture hardware, bed rails, light switches and food preparation surfaces can help limit microbial infections in hospitals and food dispensing organizations. Michels, et al. [12] show that increasing the copper content of alloys increases antimicrobial effectiveness. The contact killing is so rapid that the production of protective biofilms is not possible [13].

The specific mechanism by which copper affects cellular structures is not yet proven, but the active agent of cell destruction is generally considered to be the copper ion [11,14,15].

Recent studies showed that large amounts of copper ions were taken up by E. coli over 90 min, when cells were applied to copper coupons via an aqueous suspension (a standing drop). When cells were plated on copper using minimum liquid and a drying time of 5 seconds, the accumulation of copper ions by cells was even more dramatic, reaching a high concentration in a fraction of the time. The copper ion level of cells remained high throughout the killing phase, suggesting that cells become overwhelmed by their intracellular copper [15]. The grain structure of the copper material affects ion diffusion and hence affects bacterial destruction by copper ions.

The US Environmental Protection Agency (EPA) registers five copper alloys with public health claims [16]. All of the alloys have minimum nominal copper concentrations of 60%. Registration of copper and certain copper alloys such as brass and bronze means that the EPA recognizes these solid materials’ antimicrobial properties. Products made from any of the registered alloys are legally permitted to make public health claims relating to the control of organisms that pose a threat to human health. Laboratory studies conducted under EPA-approved protocols have proven copper’s ability to kill, within 2 hours of contact time, more than 99.9% of the following disease-causing bacteria: *Staphylococcus aureus, Enterobacter aerogenes, Escherichia coli* O157:H7, *Pseudomonas aeruginosa,* Vancomycin-resistant *Enterococcus faecalis* (VRE) and MRSA.

### Copper surface generation

In order to make use of the antimicrobial ability of copper, surfaces that contact skin and foods should be composed of copper or copper alloy. This can be accomplished with solid copper equipment or by means of copper surface coating. In general, cost considerations favour copper coatings over solid structural copper. Various metal spray techniques are available for the purpose of depositing a copper surface onto implements that can transmit microorganisms, and it is desired to identify an optimal deposition method. Accordingly, three metal spray techniques are evaluated with respect to the anti-microbial activity of the copper surfaces produced by each.

### Plasma spray

**Figure 1:**
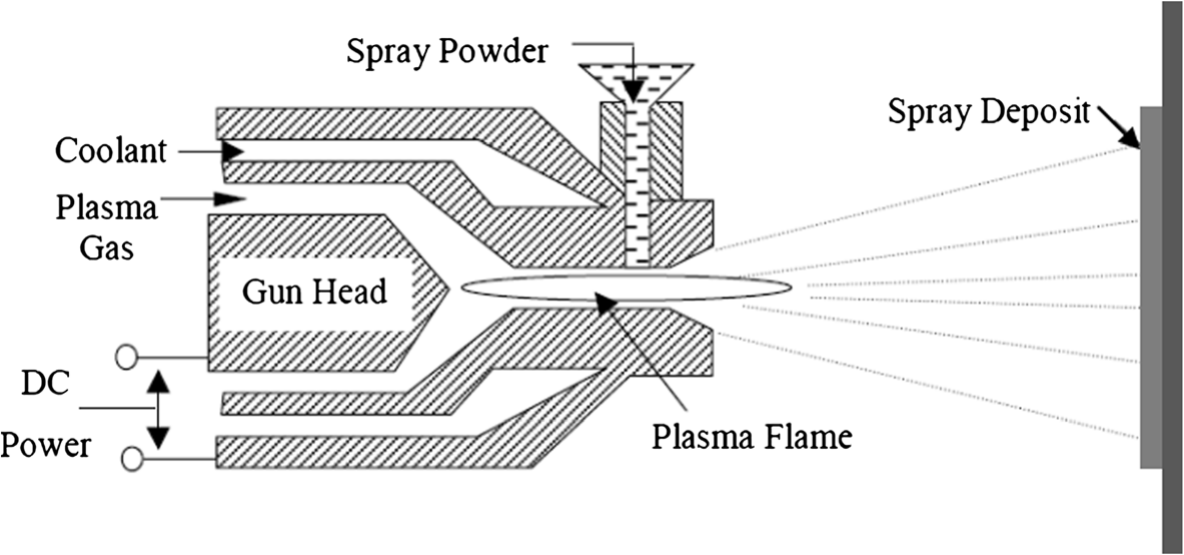
The plasma spray deposition system.

The plasma spray process shown in Figure 1 uses a DC electric arc to generate a stream of high temperature ionized plasma gas, which acts as the spraying heat source. The coating material, in powder form and carried by an inert gas, is injected into the plasma jet where it is melted and propelled towards the substrate.

The plasma spray gun includes a copper anode and tungsten cathode, which are both water cooled. Plasma gas (argon, nitrogen, hydrogen, helium) flows around the cathode and through the anode, which is shaped as a constricting nozzle. The plasma, containing suspended metal droplets, exits in the anode nozzle and is directed toward a surface, where the particles deposit.

### Arc spray

**Figure 2:**
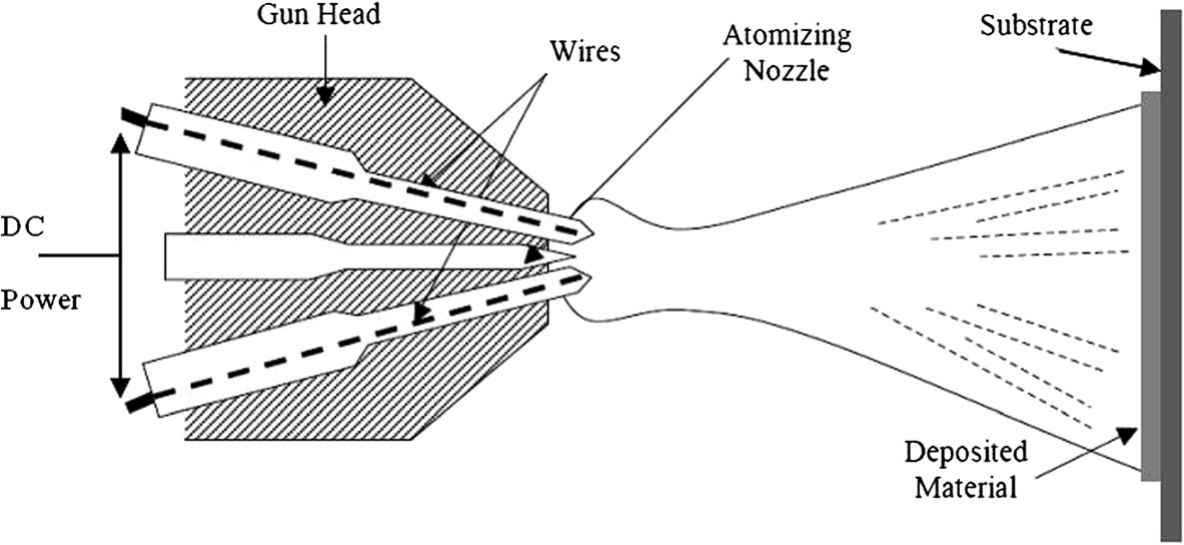
The wire Arc spray deposition system.

The arc spray process shown in Figure 2 creates an arc between two metallic wires acting as consumable electrodes. A DC voltage is applied between the wires, and an arc discharge is created at the contact of the wires. The wire electrodes are melted by the electric arc and a compressed air jet disperses the molten droplets and propels them onto a surface.

### Cold spray

**Figure 3:**
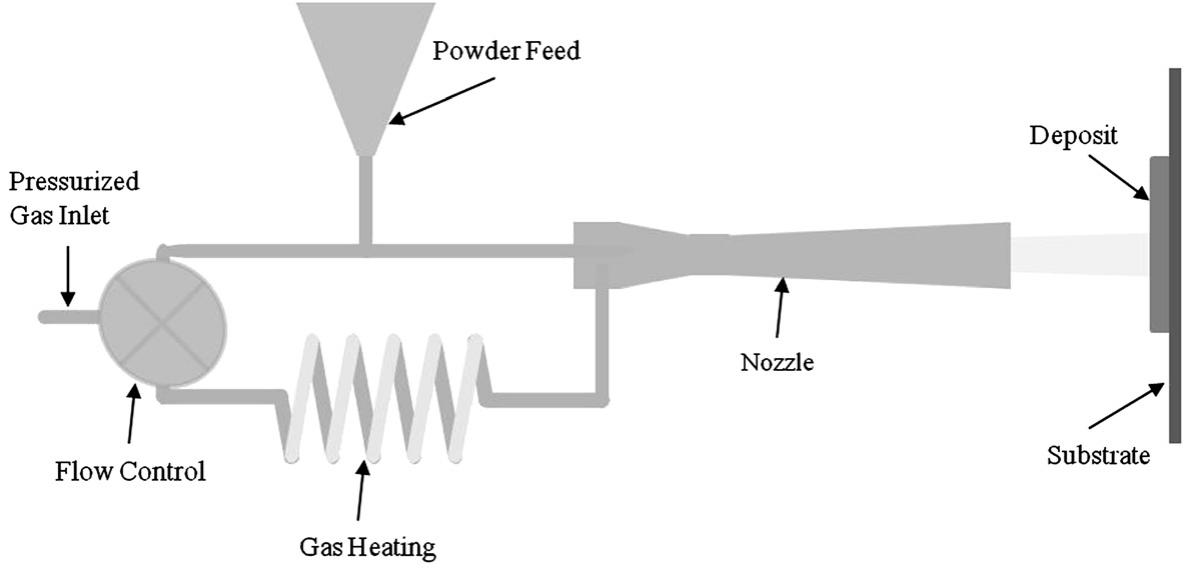
The cold spray deposition system.

The cold spray process shown in Figure 3 imparts supersonic velocities to metal particles by placing them in a heated nitrogen or helium gas stream that is expanded through a converging–diverging nozzle. The powder feed is inserted at high pressure at the nozzle entrance. The particles, entrained within the gas, are directed towards a surface, where they are embedded on impact, forming a strong bond with the surface. The term “cold spray” has been used to describe this process due to the relatively low temperatures (100-500°C) of the expanded gas stream that exits the nozzle. Subsequent spray passes increase the structure thickness. The adhesion of the metal powder to the substrate, as well as the cohesion of the deposited material, is accomplished in the solid state.

The relatively low porosity of the cold spray coating results from particle packing caused by high velocity impact. Another characteristic of high velocity impacts is the creation of grain dislocations and work hardening. The low oxide content of cold sprayed deposits occurs because the particle temperature remains low and thus inhibits oxidation.

The spray techniques described each produce impacting metal particles in distinct temperature and velocity ranges. These temperatures and velocities create metal coatings with different characteristics with respect to the presence of oxides, porosity, grain dislocations, and hardness. Because of these metallurgic differences, it is reasonable to assume that the coatings will exhibit differences in antimicrobial efficiency. Table 1 gives the particle temperatures and impact velocities, as well as the porosity and oxide ranges of the resulting deposits.

**Table 1:** Typical spray gun operating parameters

**Spray/Property**	**Temperature °C**	**Velocity, m/s**	**Porosity, %**	**Oxides, %**
Plasma	2500 - 3500	100 - 300	1 - 10	1 - 3
Wire Arc	2500 - 3500	50 - 100	5 - 20	10 - 20
Cold Spray	100 - 500	600 - 1000	<1	<1

### Test procedure

**Figure 4:**
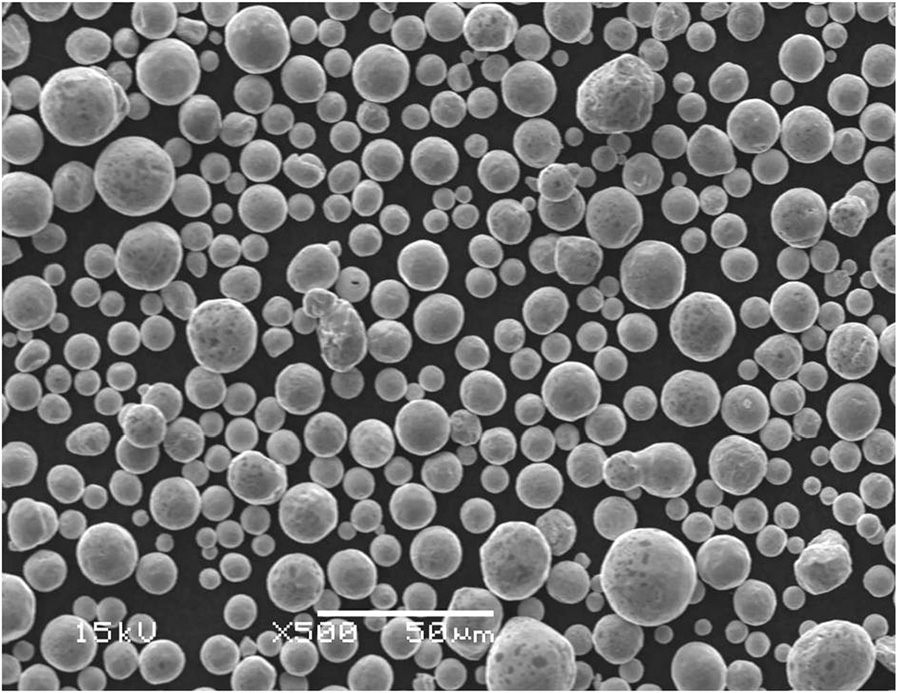
Feedstock copper powder for plasma and cold spray.

**Figure 5:**
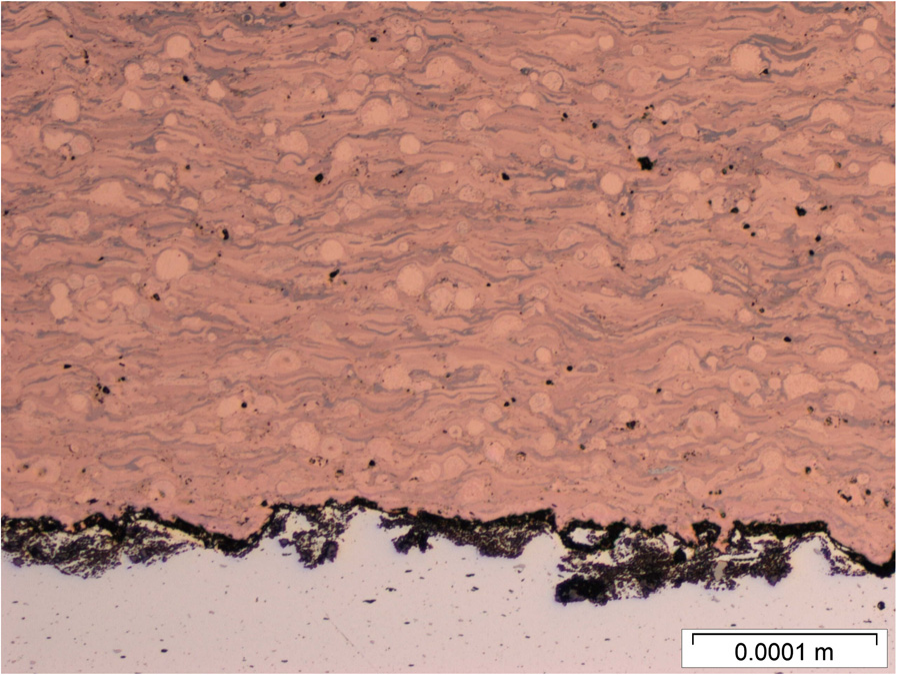
Cross sectional view of the plasma sprayed copper deposit.

**Figure 6:**
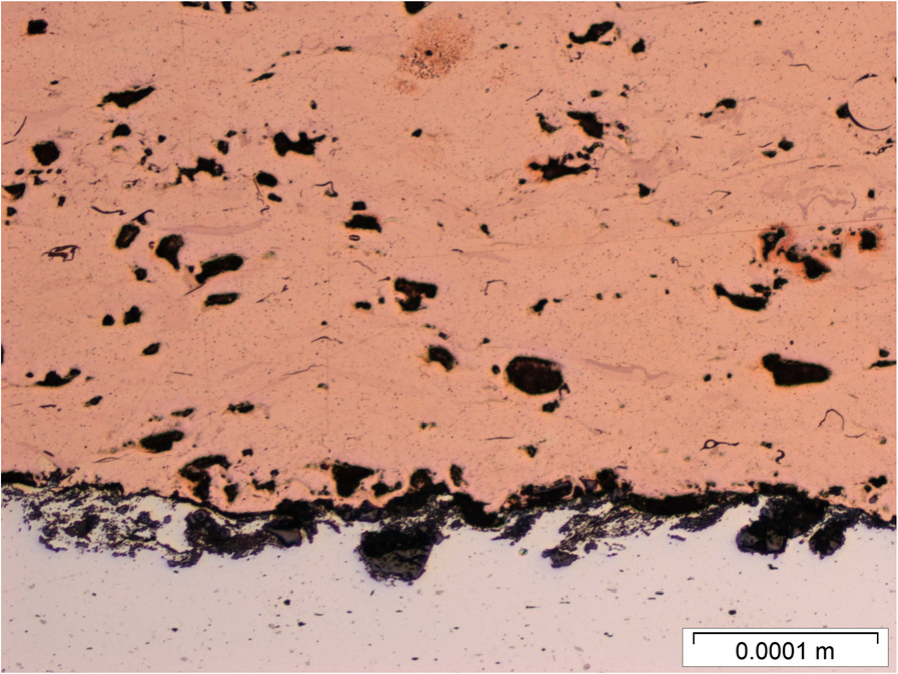
Cross sectional view of the wire arc sprayed copper deposit.

**Figure 7:**
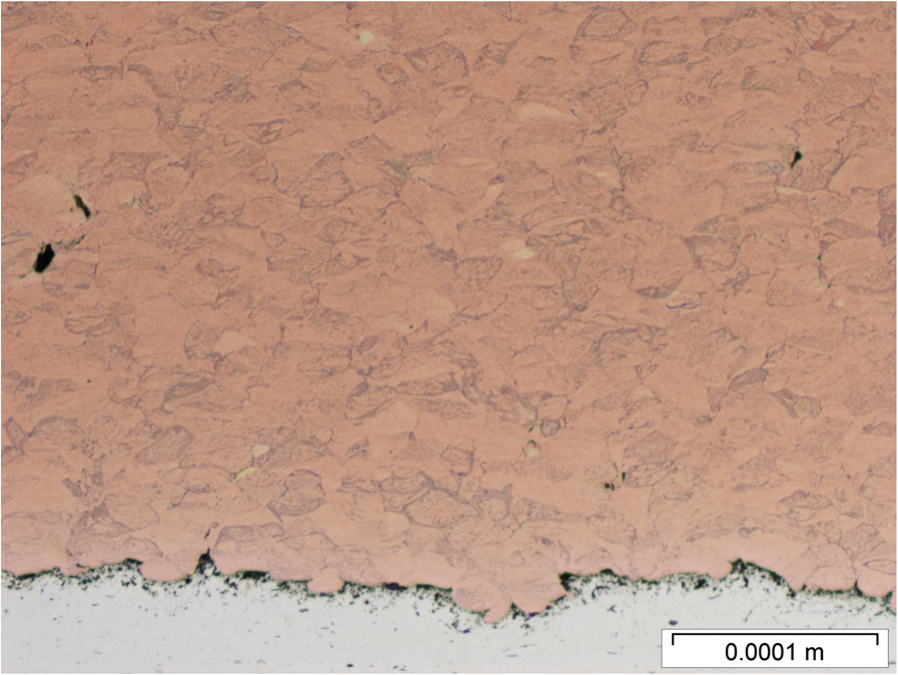
Cross sectional view of the cold sprayed copper deposit.

These three surface coating techniques were used to produce copper-coated metal coupons. Approximately 1 mm thick coatings were applied to aluminum substrates. The coatings completely covered the metal substrates with an impervious seal. The copper powder feedstock used for the plasma and cold sprays is shown in Figure 4. Cross sections of the coupons produced by the three spray techniques are shown in Figures 5, 6 and 7. Differences in microstructure are clearly evident, suggesting that differences in biological activity may also occur. Evidence of particle melting is clear for the high-temperature plasma and wire arc processes. The incidence of large voids is seen in the wire arc process cross section.

The coated coupons were inoculated with MRSA. The plated samples were then held at room temperature for two hours, after which survivors were resuspended and cultured. This procedure followed the EPA Protocol [17] “Test Method for Efficacy of Copper Alloy Surfaces as a Sanitizer”. The details of this procedure are given below [17-20].

### Carrier surfaces and preparation

The copper coupon surfaces were utilized as the test carriers and stainless steel squares were used as control carriers. The carriers were dipped into ethanol, rinsed with deionized water, and allowed to air dry. The carriers were autoclaved prior to use in test. After sterilization, each carrier was placed into a Petri dish matted with two pieces of filter paper.

### Preparation of test organisms

Ten (10) mL tubes of Synthetic Broth were inoculated from stock cultures and incubated for 24 hours at 36°C. Using a 4-mm inside diameter disposable sterile plastic transfer loop, at least three consecutive daily transfers of cultures were made in Synthetic Broth prior to use as test inoculum. Two loopfuls of culture were transferred to 10 ml broth medium and incubated for 48 hours.

The culture was thoroughly mixed on a “vortex” mixer and allowed to settle. The upper two thirds of this suspension was used as the inoculum for testing.

### Addition of organic soil load

An organic soil load containing Triton X-100 (to aid in spreading of the inoculums) was added to the test culture. A 0.25 ml aliquot of fetal bovine serum and 0.05 mL aliquot of 1% Triton X-100 was added to 4.70 ml of culture to yield a 5% fetal bovine serum and 0.01% Triton X-100 soil load.

### Inoculation of carriers

Each test and control carrier was inoculated at staggered intervals with 0.02 ml of 48 hour culture using a calibrated pipettor. The inoculum was spread to within 3 mm of the edges of the carrier. The lids of the Petri dishes were replaced and the carriers were held at room temperature (20°C) for 2 hours. The exposure period began immediately after inoculation.

### Neutralization and subculture

Following the 2 hour exposure, the carriers were transferred to jars containing 20 mL of Letheen Broth + 0.07% Lecithin + 0.5% Tween 80 at staggered intervals. Each neutralizer jar was sonicated for five minutes to suspend any survivors and rotated to mix. Serial dilutions (10^0^ – 10^-4^) of the neutralized solution from each of the jars were prepared. One (1.0) mL aliquots of those dilutions were plated in duplicate using standard spread plate technique onto sheep blood agar plates (BAP).

### Incubation and observation

The plates were incubated at 36°C for 44 hours prior to observation and enumeration. Following incubation, the plates were visually enumerated. Subcultures containing 30–300 colonies were used for calculations.

## Results and discussion

**Figure 8:**
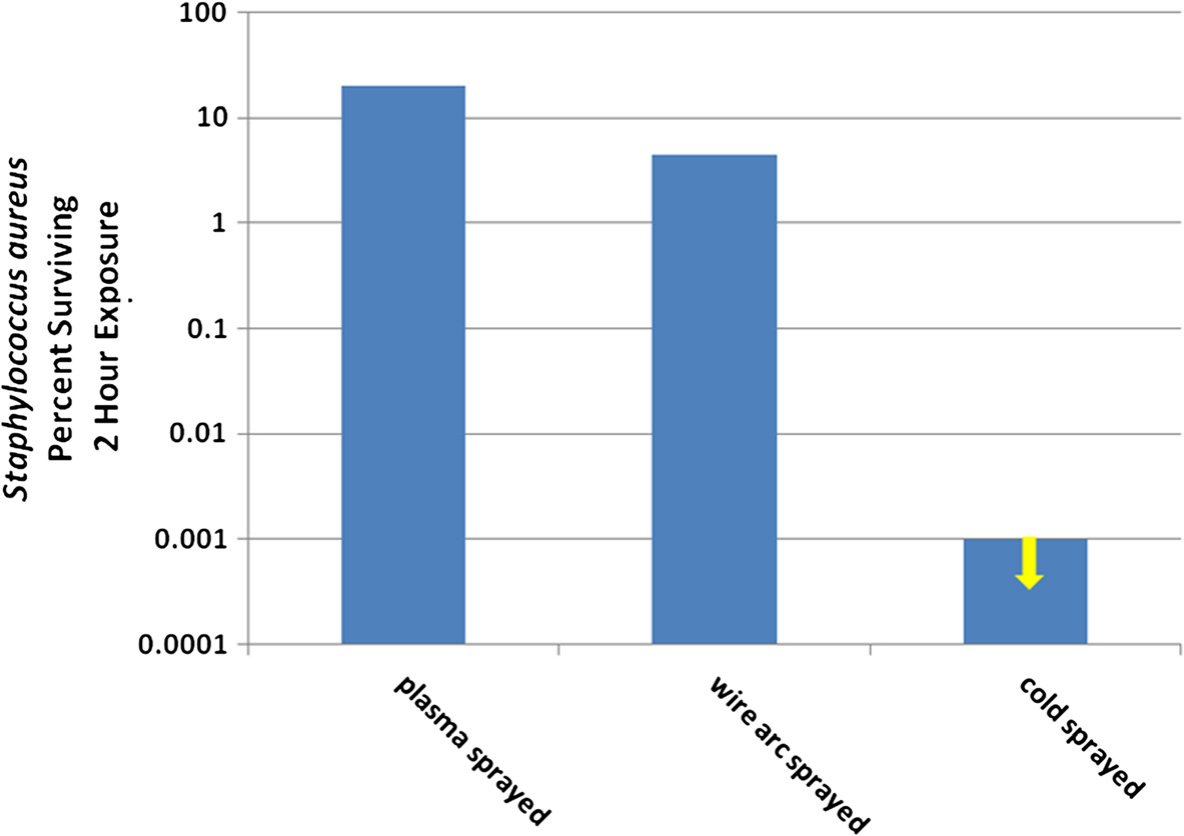
Percent MRSA surviving after exposure to various copper deposits.

The reduction of inoculated *S. aureus* was normalized by the results of the control exposure to a stainless steel surface. The results of these tests in terms of the percent of surviving *S. aureus* after two hours are shown in Figure 8. The result for cold spray was below minimal measurement thresholds and is thus reported as “less than”.

The results show a greater than three order of magnitude difference in kill efficiency between the plasma and wire arc methods and the cold spray method of copper deposition. This large difference in anti-microbial effectiveness between copper spray deposition methods requires an examination of how the deposition mechanism affects the nature of the copper. The plasma and wire arc methods deposit molten particles at relatively low velocity (<200 m/s). The cold spray method deposits solid particles at high velocity (>600 m/s). Champagne, et al. [21] have shown that the high velocity impacts of cold sprayed particles lead to extreme work hardening and correspondingly high dislocation density within the deposit. For example, the Vickers Hardness values for plasma, wire arc, and cold spray deposited copper were 94, 105, and 141, respectively. Ion diffusion in metals is augmented by the presence of grain dislocations, known as “pipe diffusion”, and ionic diffusion occurs principally through these dislocations. The relationship between dislocation density and Vickers Hardness is [22] ρ **∝** H^2^, and the relationship between diffusivity and displacement density is given by [23] D_p_**∝** ρ. Diffusivity in metals thus varies as the square of hardness and is therefore very sensitive to impact hardening by cold spray deposition. The diffusion of copper ions can therefore be significantly increased through the hardness increase produced by the cold spray process, which serves to enhance the flow of Cu^2+^ ions needed for microbial destruction.

## Conclusions

The effectiveness of copper and copper alloys as anti-microbial coatings on touch-surfaces has been well documented by many researchers [5-7,10,12-15]. Except for the copper content of alloys, the effects of the metallurgical properties of the copper coatings have not been investigated by these efforts. The significant anti-microbiologic differences between coatings produced by different spray techniques, as shown here, demonstrate the importance of the copper application technique and of the resulting deposition structure. The cold spray technique showed superior anti-microbial effectiveness caused by the high impact velocity imparted to the sprayed particles which results in high dislocation density and high ionic copper diffusivity.

**Figure 9:**
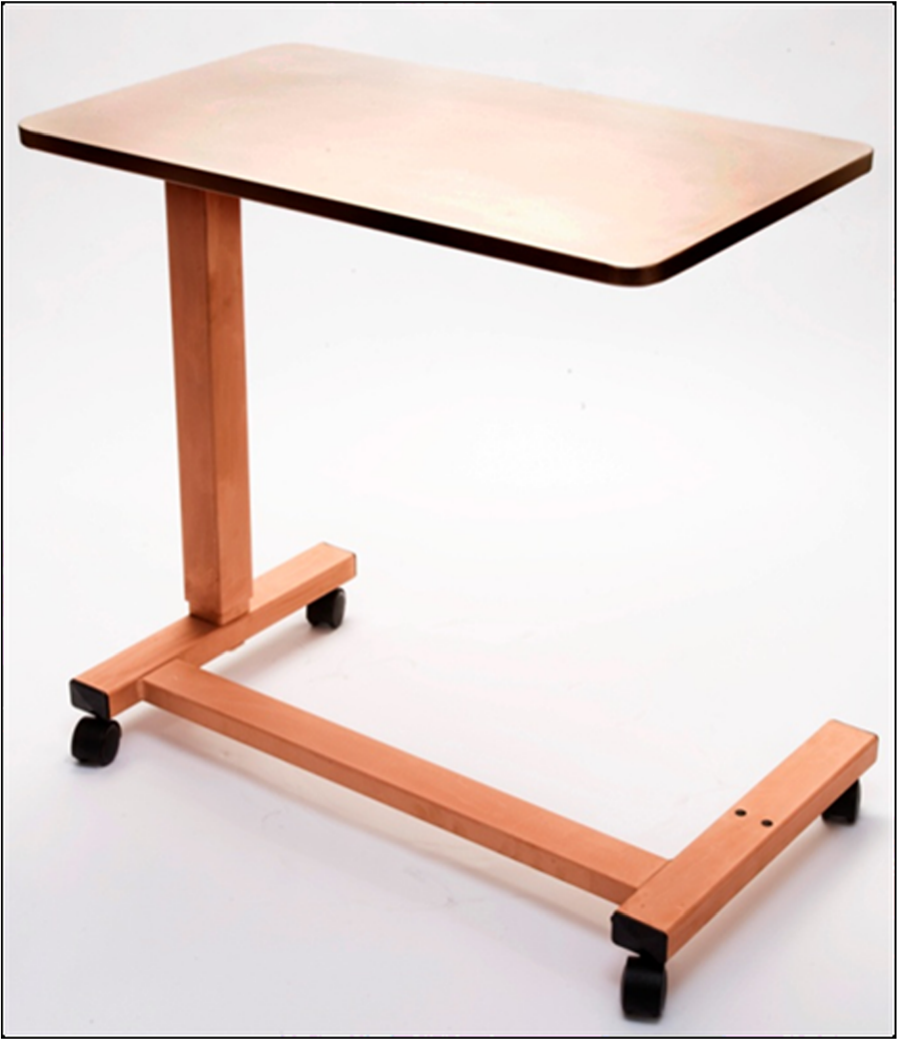
Hospital table coated with cold-sprayed copper.

The cold spray process is a mature technology which is currently in use for a variety of applications requiring various metal coatings. The cold spray process can readily apply copper coatings onto touch surfaces. Figure 9 is an example of copper coating by cold spray. The hospital tray and the entire metal support structure of the hospital table have been coated with cold-sprayed pure copper. In addition to providing highly efficient antimicrobial surfaces, the cold spray technique is less likely to damage heat sensitive substrates than are high temperature thermal sprays. This work is a proof-of-concept effort, and additional, more statistically significant, work should be performed in order to justify commercialization.

## Competing interests

The authors declare that they have no competing interests.

## Authors’ contributions

VKC conceived and directed the test program. DJH interpreted the test results and justified the mechanism. Both authors read and approved the final manuscript.
